# 

**Published:** 1856-06

**Authors:** 


JULY, 1856.

THE

### Dental Register

### OF THE WEST.

JAMES TAYLOR, M. D., D. D. S., Editor.

Published Quarterly at $3 per annum.

CINCINNATI:

T. WRIGHTSON & CO., PRINTERS,

167 Walnut Street.

